# Natural Small Molecules in Gastrointestinal Tract and Associated Cancers: Molecular Insights and Targeted Therapies

**DOI:** 10.3390/molecules27175686

**Published:** 2022-09-03

**Authors:** Fahadul Islam, Saikat Mitra, Talha Bin Emran, Zidan Khan, Nikhil Nath, Rajib Das, Rohit Sharma, Ahmed Abdullah Al Awadh, Moon Nyeo Park, Bonglee Kim

**Affiliations:** 1 Department of Pharmacy, Faculty of Allied Health Sciences, Daffodil International University, Dhaka 1207, Bangladesh; 2 Department of Pharmacy, Faculty of Pharmacy, University of Dhaka, Dhaka 1000, Bangladesh; 3 Department of Pharmacy, BGC Trust University Bangladesh, Chittagong 4381, Bangladesh; 4 Department of Pharmacy, International Islamic University Chittagong, Chittagong 4318, Bangladesh; 5 Department of Rasa Shastra and Bhaishajya Kalpana, Faculty of Ayurveda, Institute of Medical Sciences, Banaras Hindu University, Varanasi 221005, Uttar Pradesh, India; 6 Department of Clinical Laboratory Sciences, Faculty of Applied Medical Sciences, Najran University, P.O. Box 1988, Najran 61441, Saudi Arabia; 7 Department of Korean Medicine, Kyung Hee University, 26 Kyungheedae-ro, Dongdaemun-gu, Seoul 05254, Korea

**Keywords:** natural products, cancer, gastrointestinal tract, small molecules, apoptosis

## Abstract

Gastric cancer is one of the most common cancers of the gastrointestinal tract. Although surgery is the primary treatment, serious maladies that dissipate to other parts of the body may require chemotherapy. As there is no effective procedure to treat stomach cancer, natural small molecules are a current focus of research interest for the development of better therapeutics. Chemotherapy is usually used as a last resort for people with advanced stomach cancer. Anti-colon cancer chemotherapy has become increasingly effective due to drug resistance and sensitivity across a wide spectrum of drugs. Naturally-occurring substances have been widely acknowledged as an important project for discovering innovative medications, and many therapeutic pharmaceuticals are made from natural small molecules. Although the beneficial effects of natural products are as yet unknown, emerging data suggest that several natural small molecules could suppress the progression of stomach cancer. Therefore, the underlying mechanism of natural small molecules for pathways that are directly involved in the pathogenesis of cancerous diseases is reviewed in this article. Chemotherapy and molecularly-targeted drugs can provide hope to colon cancer patients. New discoveries could help in the fight against cancer, and future stomach cancer therapies will probably include molecularly formulated drugs.

## 1. Introduction

The term “gastrointestinal cancer” refers to malignant conditions affecting the digestive organs, including the esophagus, stomach, gallbladder, pancreas, small intestine, large intestine, and rectum. Other digestive organs affected by gastrointestinal cancer include the gallbladder and intestines [1,2]. Esophageal cancer is the most frequent gastrointestinal cancer. This category contains cancers that start in the gastrointestinal tract (GI tract), cancers that begin in digestive organs other than the stomach and small intestine, and cancers that form in the GI system [3,4]. Symptoms may be directly related to the damaged area, and may include obstruction (which may cause difficulty in eating or defecation), abnormal bleeding, and other problems associated with the condition. These symptoms are directly related to the damaged organ. The organ that is being negatively affected is related to the symptoms experienced by the patient in complex ways [5]. Carcinomas of the gastrointestinal process and the organs that support it (the pancreas, liver, and gall bladder) have exceptionally high incidence and mortality rates. The prevalence of different forms of gastrointestinal cancer varies widely among regions. However, according to the typically dismal outlook for patients with GI cancer throughout this period, chemotherapeutics are often explored as a treatment option [6,7].

Consequently, it should not come as a shock that research into the cancer-preventing qualities of antioxidants has been prevalent, specifically in protecting against gastrointestinal cancers. Over the past few decades, gastrointestinal cancer has been the focus of more study and molecular discoveries than all other solid tumor types combined [8,9]. Cigarette smoking, heavy alcohol use, advanced age, certain viruses and bacteria, chronic pancreatitis, obesity, and being overweight are all conditions that raise the chances of gastrointestinal cancer. Although recent studies have focused on the molecular origin of gastrointestinal cancer, it continues to be one of the leading causes of cancer-related fatalities in developed countries [10].

Although the source and etiology of gastrointestinal malignancy may vary, poor survival after late diagnosis and metastasis is a feature shared among all these diseases. Most digestive malignancies take time to grow and, if caught early enough, can allow a better opportunity for intervention and prevention. Not unexpectedly, the introduction of various screening and diagnostic procedures aimed at early diagnosis of pre-malignant and early-stage malignancy has resulted in a steady reduction in numerous gastrointestinal cancers, including colorectal and gastric cancer. However, the currently used screening methods are ineffective and often intrusive and costly, leading to low acceptability. In light of these limitations, there is an undeniable need to develop reliable biomarkers that can be used in the early stages of human cancers [11,12].

Small molecules comprise most medications, such as insulin, aspirin, and antihistamines. Secondary metabolites, such as lipids, glycosides, alkaloids, and natural phenols, are included in this category in addition to primary metabolites such as fatty acids, glucose, amino acids, and cholesterol [13]. Other examples include natural phenols and diterpenoids; for instance, sugiol is a potential leading chemical in the treatment of human gliomas because it prevents the proliferation of glioma cells [14]. It can easily penetrate the cell membrane and enter the cytoplasm, potentially interacting with the molecules housed within. Enzyme inhibitors are chemicals that stop enzymes from performing their normal function [15]. They impede the action of essential enzymes, preventing the transmission of signals required for developing cancer cells. In this way, blocking these cell signals can stop the progression of cancer and its ability to metastasize [9,16]. Small compounds can be used as medicines to silence proteins in cancer cells. These small molecule inhibitors shut off protein pathways, reducing cancer cell growth and proliferation. As one of the most common and distinct malignancies, gastric cancer (GC) remains a global health problem. PI3K is one of the most significant signaling pathways, regulating cell proliferation and survival in GC. The PI3K/Akt axis directs the conversion of phosphatidylinositol 4,5- bisphosphate (PIP2) to phosphatidylinositol (3,4,5)-trisphosphate (PIP3), a second messenger that then transmits a phosphoryl group to a downstream protein kinase [17]. Patients having a history of colon cancer or large adenomatous polyps before 60, inflammatory bowel illness, a family history of adenomatous polyposis, or hereditary nonpolyposis are at a higher risk of colon cancer than those with no such history. People with adenomatous polyps or colon cancer are at risk as well. The most common primary liver cancer is hepatocellular carcinoma [18]. Trans-arterial chemoembolization (TACE) is carried out through intermittent chemotherapy infusion (mainly doxorubicin and cisplatin) and subsequent vascular embolization of ischemia. Small molecule inhibitor medications that treat pancreatic cancer block the HSP90, FAK, hedgehog, Bcl-2, Mcl-1, KRAS, and Cdc37 pathways. These methods cause pancreatic carcinoma (PC) death and restrict PC proliferation and development. Epithelial gastrointestinal malignancies are the most common form; however, this paper focuses on other cell types and lymph nodes [19,20]. Biologically active chemicals for the therapy of human illnesses, including cancer, may be found in abundance in the marine environment. Marine animals evolved by different evolutionary routes than their terrestrial counterparts as a result of their ability to adapt to their unusual environmental circumstances, creating distinct compounds that are both diverse and complicated. With hundreds of novel marine natural products (MNPs) being found every year, more than 36,000 chemicals have already been extracted from marine micro- and macro-organisms, including but not limited to fungi, bacteria, microalgae, macroalgae, sponges, corals, mollusks, and tunicates. Modern pharmacology has begun to be influenced by marine-based pharmaceuticals, and several anti-cancer medications made from marine compounds have been approved for clinical use. These include cytarabine, vidarabine, nelarabine (a prodrug of ara-G), fludarabine phosphate (a pro-drug of ara-A), trabectedin, eribulin mesylate, brentuximab vedot [21]. As a first line of quick defense against pathogen invasion, insects create a vast variety of proteins and peptides. These substances have a variety of effects, including the stimulation of insect immune systems and direct contact with the target tumor cells or viruses. It has been shown that certain insect peptides inhibit the production of viral genes, proteins and rybosilate DNA, while others rupture membranes, trigger apoptosis, or halt the cell cycle. A number of insect-derived peptides have been isolated and described, and show great promise for treating major human illnesses such as leukemia, HIV, and herpes simplex virus (HSV). Several challenges must be conquered, however. Problems including peptide cytotoxicity, protease sensitivity, and high manufacturing costs have yet to be resolved [22]. Targeting glutaminase metabolism by blocking glutaminase has garnered attention as a possible cancer treatment technique, as glutaminase is critical in stimulating cell metabolism during the proliferation of cancer cells. A sequential screening of a database of traditional Chinese medicines was carried out, followed by simulations of similarity to existing drugs and molecular dynamics against the glutaminase active site. After screening the traditional Chinese medicine database against glutaminase, researchers found twelve effective compounds. This was followed by a drug-likeness filter using Lipinski and Veber rule criteria. With binding energies of 9.3 and 9.7 kcal/mol, respectively, compared to the binding energy (kcal/mol) of 6-Diazo-5-Oxo-L-(4.7 Norleucine) with glutaminase, ZINC03978829 and ZINC32296657 were shown to have greater binding energies (BE) than the control molecule. These findings were subsequently evaluated using molecular dynamics simulations, and a 100 ns MD simulation showed that the hits formed stable complexes with glutaminase and produced 2–5 hydrogen bond connections. According to this research, these compounds might be used as glutaminase inhibitors in the fight against cancer [23]. In vitro and in vivo investigations demonstrated their excellent anticancer efficacy in combination with chemotherapy and radiotherapy; the administration of this nanomedicine effectively inhibited tumor growth and greatly extended the survival rate of cisplatin-resistant A2780CIS-tumor-bearing mice. Guided by in vivo fluorescence imaging, radio-chemotherapy facilitated boosted therapeutic outcomes and minimized undesired side effects. The success of this theranostic system offers new hope that supramolecular nanomedicines can potentially be translated into clinical practice [24]. The next generation of personalized medicine, known as nanotheranostics, combines therapy and diagnosis with nanoformulations to address the challenges of accurate cancer diagnosis, logical management, and effective therapy, with the goal of significantly raising the survival rate and enhancing quality of life for cancer patients. Supramolecular cancer nanotheranostics provide unmatched benefits in early-stage diagnosis and personalized treatment, in contrast to most traditional platforms, which have subpar theranostic capabilities; as such, they have great prospects in clinical translations and applications [25]. A particularly interesting contender in this area is supramolecular chemotherapy, which integrates non-covalent interactions with conventional chemotherapy and may be utilized effectively for targeted drug delivery. Difficulties with standard chemotherapy for clinical applications can be successfully overcome using supramolecular chemistry [26].

The aim of this review is to outline the molecular pathogenesis of gastrointestinal cancer and the role of natural small molecules in preventing gastrointestinal cancers. Additionally, synergistic effects among natural small molecules as well as clinical evidence that can be applied to the treatment of gastrointestinal cancers are highlighted as well.

## 2. Methodology

**Figure 1:**
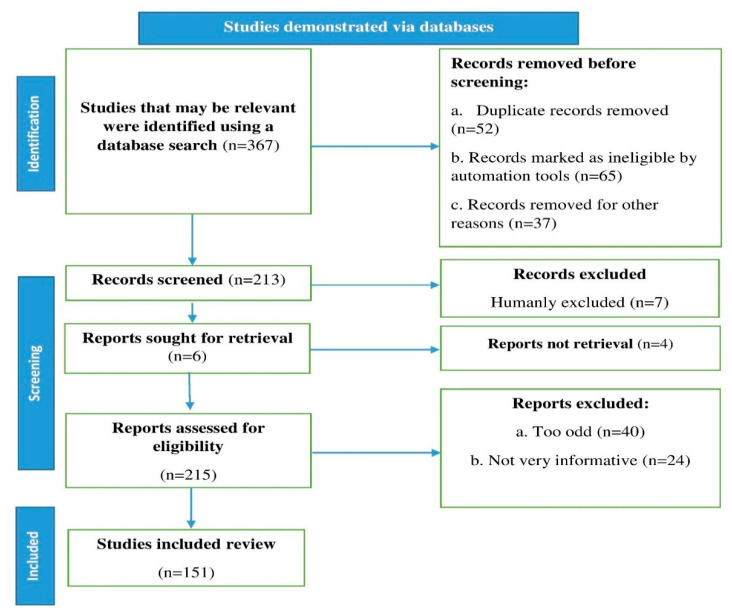
A flow chart illustrating the required steps when choosing published data to be used in the current study; n = number of literature reports.

The following databases were used: PubMed, Scopus, and Web of Science, with the keywords natural small molecules, gastrointestinal cancer, synergistic effects, and clinical evidence. English-language research reports, reviews, and original research articles up until 2022 were chosen and examined. According to the guidelines of Page et al. [22], an algorithm was utilized that followed the flowchart in Figure 1 and included all of the processes and requirements for selecting the necessary literature.

## 3. Molecular Pathogenesis of Gastrointestinal Cancer

The development of colorectal cancer is caused by the gradual accumulation of genetic and epigenetic changes which ultimately result in the transformation of normal colonic mucosa into adenocarcinoma in the affected area of the body. Approximately 75 percent of colorectal cancers are sporadic, meaning they arise in individuals who do not have a genetic predisposition for colorectal cancer or a family history of the disease. Sporadic cases of colorectal cancer have been divided into three primary categories according to the genes in which mutations are most commonly prevalent. These genes have been discovered through linkage studies on cancer-prone families and individual mutation analyses on candidate genes chosen on the basis of functional data. Recent studies have identified a huge number of genes that rarely undergo mutations in addition to those that experience mutations on a more frequent basis. These genes are likely only documented in a small number of pathways due to their low rate of mutation. Gene-oriented models of the development of colorectal cancer are being phased out in favor of pathway-oriented models [27]. Pancreatic cancer is a disease with a very low treatment success rate, with just a 5% probability of survival after five years. Studies of both sporadic and hereditary pancreatic cancer have now provided a better understanding of the molecular basis of this disease. A number of anomalies have been uncovered, elucidating the genetic mutations that occur in pancreatic cancer. Many of these anomalies have an influence on the development and evolution of pancreatic cancer, ranging from severe chromosomal abnormalities to point mutations. The discovery of precursor lesions within pancreatic ducts led to the construction of a pancreatic cancer progression model and the identification of the early-stage and late-stage abnormalities that eventually lead to invasive disease. Understanding the genetic pathways that contribute to the development of pancreatic cancer may prove to be useful tool in screening, treatment, and prevention of the disease [28]. Stomach cancer is the fifth most common kind of cancer and the third leading cause of cancer deaths globally. Gastric cancer is a complicated illness that arises from a combination of environmental and host-related variables. The late clinical manifestation of the disease, and its underlying biological and genetic variability all play a role in the high fatality rates observed in gastric cancer. Improving patient outcomes requires a thorough understanding of the various genetic disorders linked to this malignancy. In recent years, scientists have made significant progress in understanding the molecular landscape of stomach cancer. This includes the discovery of novel molecular components related to gastric malignancy and development, such as ARID1A and RHOA, as well as cellular pathways and tissue populations. The completion of the Cancer Genome Atlas (TCGA) project marked a turning point in the molecular characterization of gastric cancer [29].

## 4. Natural Small Molecules in Gastrointestinal Cancer

### 4.1. Gastric Cancer

**Figure 2:**
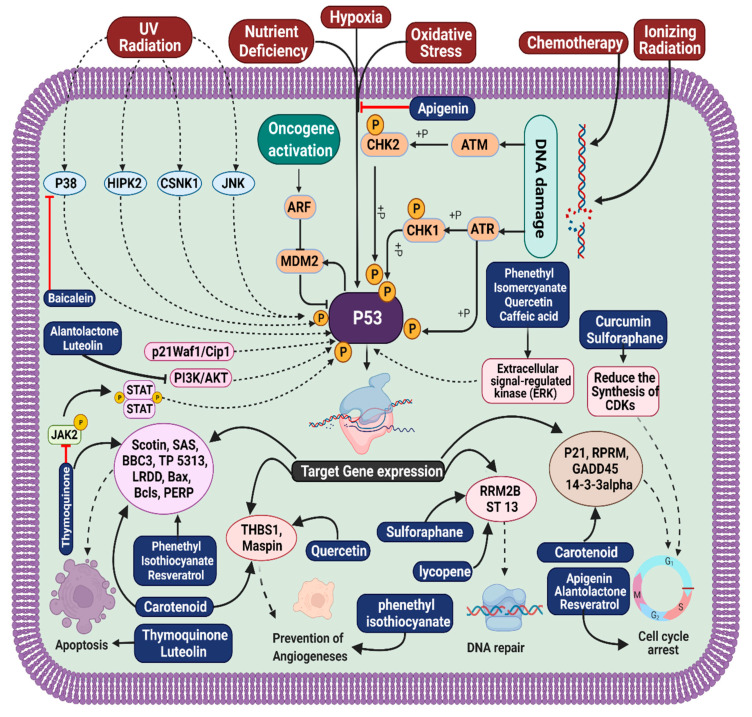
Illustration representing the probable blockade of sites by small molecules in the gastrointestinal tract and associated cancers.

Stomach cancer is one of the most common types of cancer; people die globally from it at an alarming rate. Resveratrol is widely recognized for its anti-inflammatory and antioxidant capabilities as well as its potential to inhibit both platelet aggregation and the growth of a variety of cancer cells. In several murine models of human malignancies as well as in UV-B and chemo-induced murine skin cancer, the molecule’s potential chemopreventive and chemotherapeutic properties have been recorded in detail. Furthermore, it has been shown to have chemopreventive and chemotherapeutic properties in all three phases of carcinogenesis (initiation, promotion, and progression). Numerous in vitro and in vivo studies have shown this compound’s ability to alter a broad range of signaling pathways and targets that are associated with carcinogenesis and cancer progression [30]. The flavonoid molecule quercetin has been proven to have anti-inflammatory, anti-cancer, and anti-ulcer properties; in addition, the drug protects against colon cancer through the induction of apoptosis in animal models and prevents the growth of a range of cancer cells (Figure 2).

In addition, two investigations have indicated that quercetin protects mucosal tissue against ulcer-induced damage. Quercetin’s anti-ulcer action has been found to be associated with a reduction in the growth of *Helicobacter pylori* [31]. Apigenin is a flavonoid that is found in abundance in nature. Apigenin-rich vegetable celery is utilized medicinally in several Asian nations for a range of inflammatory disorders. The anti-inflammatory properties of apigenin have been studied both in vitro and in vivo. The modulation of NF-kB activation and suppression of COX-2, iNOS and the proinflammatory cytokines interleukin IL-1β, IL-6, and IL-8, along with the downregulation of tumor necrosis factor production, are among the mechanisms relevant to apigenin’s activity. The molecule’s anti-gastric cancer action might be attributed to the compound’s ability to halt cell growth and induce cell death. A range of bioactivities of this molecule have been studied, including those that inhibit platelet aggregation, counteract oxidation, and reduce *Helicobacter pylori* activity. The double bond of apigenin’s flavonoid skeleton at C2 and C3 and hydroxylation at the A and B rings are most likely responsible for its bioactivity. In addition, apigenin has the ability to naturally absorb free radicals [32]. The anticancer properties of the cruciferous vegetable constituent phenethyl isothiocyanate (PIC) have recently been brought to light. Chemopreventive and chemotherapeutic medications for the treatment and prevention of cancer in humans could be made with the help of this compound. Research shows that PIC may inhibit human gastric cancer cells from proliferating and migrating by blocking ERK1/2, PKC/NF-kB, and other signaling pathways in human stomach tissue [33]. Sulforaphane is another isothiocyanate molecule, and is found in many cruciferous vegetables, such as broccoli, brussels sprouts, and cabbage. Research on sulforaphane has demonstrated that it has noteworthy biological effects, including those that combat oxidation, inflammation, ageing, and germs. It has been claimed that sulforaphane has considerable anticancer effects on cancer cells via the suppression of cell proliferation, promotion of apoptosis, prevention of metastasis, and cellular anti-angiogenesis properties [34]. Sesquiterpene lactones such as alantolactone are produced from the plant *Inula helenium* L. Anti-bacterial, anti-fungal, anti-inflammatory, anti-proliferative, and anticancer activities of this compound have been found in studies of its neuroprotective and anti-inflammatory activities. Several malignancies, including breast cancer, lung cancer, colon cancer, liver cancer, and cervical cancer, have been shown to be affected by alantolactone. The potential of alantolactone to suppress cancer cell migration in breast and lung cancer has been identified by researchers [35]. Baicalein, one of the principal flavonoids in *Scutellaria baicalensis,* has been traditionally employed for its anti-inflammatory and anticancer effects. A reduction in cell migration and motility may be caused by baicalein’s inhibition of the p38 signaling pathway. Because of this, baicalein acts as a local and systemic inhibitor of the spread of stomach cancer cells. Baicalein may therefore be useful in the treatment of stomach cancer as a therapeutic medication [36].

Thymoquinone is one of the active constituents in black cumin seed oil, and is thought to be responsible for many of its medicinal properties. There is evidence that thymoquinone causes apoptosis and promotes treatment resistance in prostate cancer cells, colorectal cancer cells, pancreatic cancer cells, and breast cancer cells. The development of various tumor cells is slowed by thymoquinone, including colorectal carcinomas, pancreatic carcinomas, and breast adenocarcinomas. However, thymoquinone had no impact on the phosphorylation of STAT3, only STAT5. Thymoquinone’s ability to reduce c-Src and JAK2 activity has been linked to a decrease in STAT3 activation. Bcl-2, cyclin D, survivin, and vascular endothelial growth factor (VEGF) were all downregulated by thymoquinone, activating caspase-3,7,9 in the process. This is possible because thymoquinone decreases STAT3 gene expression. This compound has shown success in mice with xenograft tumors [37]. Tomatoes, watermelons, guavas, and pink grapefruit all contain a pigment called lycopene, which is responsible for their reddish-orange hue. It has considerable antioxidant properties compared to carotene and tocopherol. According to epidemiological studies, high dietary lycopene intake has been linked to a decreased risk of stomach cancer. By reducing redox-sensitive signaling pathways, lycopene decreases oxidative damage to DNA in human prostate, breast, and hepatocellular carcinoma cell lines. MAPK and NF-kB pathways are two of the pathways that make up this group. Additionally, lycopene has been shown to protect DNA from oxidative damage in mice. Reactive oxygen species (ROS) and the catenin-c-myc/cyclin D1 axis are responsible for causing apoptosis by reducing ROS levels. Because it prevents beta-catenin from moving into the nucleus and the synthesis of vital genes for cell survival, lycopene induces apoptosis in stomach cancer cells [38]. The frequency of nuclear expression of NF-kB in gastric cancer tissues was substantially higher than the frequency of nuclear expression in nonmalignant gastric tissues. Infection with *H. pylori* boosted the production of MMP-9, IL-1, and IL-8 in AGS cells, as well as the activation of NF-kB, which stimulated this activity. A large rise in reactions may be reversed by treatment with caffeic acid. Tumor development and spread may be inhibited by caffeic acid as well [39].

Allicin, a molecule found in garlic, has been revealed to have anticancer properties when tested in vitro. For over 3000 years, garlic has been consumed and utilized to treat a wide range of ailments. Garlic’s pungent flavor makes it a popular spice. Recent epidemiological studies demonstrate that increasing consumption of allium-containing foods such as garlic may lower the chance of acquiring certain types of cancer. Garlic’s principal biologically active component is allicin, known as diallyl thiosulinate. The antibacterial and antifungal properties of this compound were reported by C.J. Cavallito in 1944, who was the first person to isolate and experiment on it. S-allyl-L-Cysteine Sulfoxide (alliinase) is the enzyme that generates allicin from this substrate. Alliin can only exist as a racemic enzyme, as it is chiral. Diallyl disulfide can be oxidized to produce this racemic form of the chemical. Garlic cloves contain alliinase and alliin in separate compartments, and undamaged cloves do not contain allicin. Alliin and alliinase interact with the garlic clove when it is crushed, releasing allicin, pyruvic acid, and ammonia [40].

Luteolin (3′,4′,5′,7′-tetrahydroxyflavone) is a kind of flavonoid found in a wide variety of fruits, vegetables, and medicinal plants [41]. As a traditional Chinese medicine, luteolin may cure a wide range of ailments, including inflammatory disorders such as cancer and hypertension [42]. More than a dozen studies have shown that luteolin inhibits tumor development by several methods, including anti-proliferation, pro-apoptosis, anti-metastasis, and angiogenesis [43,44].

The pharmacological effects of phenolic compounds and terpenoids, which are key components present in nutrient-rich fruits, vegetables, and spices, include the capacity to combat cancer. According to certain findings, terpenoids with different amounts of isoprene units may have anticancer properties. Many terpenoids that are currently used therapeutically have been studied in depth. According to previous studies, natural terpenoids have been shown to have cytotoxic effects on a variety of tumor cells. As a result of this research, the notion that plant-derived pharmaceutical substances can effectively inhibit NF-kB signaling, which plays an important role in the genesis of cancer, has gained more credibility [45].

**Figure 3:**
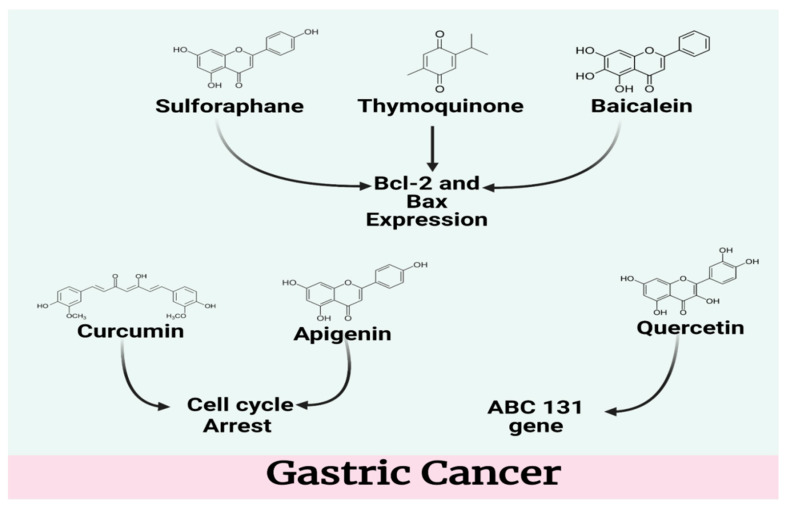
Illustration representing the probable sites blockaded by small molecules in gastric cancer.

Green tea contains Epigallocatechin-3-gallate, or EGCG, which is the most active and abundant component found in plant-based preparations. Green tea’s health advantages are largely thanks to this compound. EGCG has long been known to decrease both cell proliferation and apoptosis (Figure 3). EGCG is able to limit the growth of tumors via a variety of mechanisms, including suppression of proliferation and activation of apoptosis. Reduced tumor growth in mice has been observed as a result of EGCG’s anti-angiogenic effect and its ability to suppress angiogenesis. Angiogenesis, the production of new blood vessels from pre-existing capillaries, is required for the development and subsequent spread of solid tumors. Angiogenesis is triggered by tumor cell release of particular angiogenic substances [46]. In many cancer cells, the thioredoxin reductase (TrxR) 1 enzyme is often overexpressed. TrxR1 is a promising target for the therapy of cancer, as it slows the growth and spread of tumors. Previous studies have shown that the curcumin derivative B19 might activate ER stress and cause cancer cells to undergo apoptosis. B19′s molecular target and upstream mechanism are as yet unknown, however. The results of the aforementioned study show that B19 directly suppresses TrxR1 enzyme activity, which increases oxidative stress and generates ROS-mediated ER Stress and mitochondrial malfunction, which ultimately causes cell cycle arrest and death in human gastric cancer cells. According to computer-aided docking, B19 might engage the TrxR1 protein by forming a covalent connection with the residue Cys-498. The anti-cancer effects brought on by B19 were completely undone by blocking ROS generation. It was found that this naturally derived chemical compound has the ability to target the biomarkers associated with gastric cancer and act as a potential pharmacological agent [45]. Table 1 provides an overview of potential compounds for treatment of gastric cancer.

**Table 1:** Natural small molecules in gastric cancer.

Name of Compound	Study Model	Dose	Results	Mechanism of Action	References
Resveratrol	In vitro (HP Strains)	6.25 µg to 100 µg	MIC50 and MIC90 were 12.5 and 25 µg	Inhibited the replication of *H. pylori*	[47]
	In vitro (gastric adenocarcinoma SNU-1 cells)	10 and 100 μM	Apoptosis	Decreased protein kinase C activity, promoted cell cycle arrest, and reduced gastric cancer cell growth induced by nitrosamine.	[48]
Quercetin	In vitro (Parental EPG85-257P cell line)	12 μM	Induced apoptosis	Downregulation of ABCB1 gene	[49]
	In vitro (AGS cells)	0, 10, 20, 40, 80, 160, and 320 μM	Induced apoptosis	Quercetin promoted morphological alterations and lowered vitality	[50]
Apigenin	In vitro (HGC-27 and SGC-7901 cells)	10 μg/mL	Induced Apoptosis	Suppressed the growth of stomach cancer	[51]
	In vivo (Mongolian gerbils)	30–60 mg/kg/day		Mongolian gerbils with atrophic gastritis and dysplasia/gastric cancer had their cancer rates considerably reduced	[32]
isothiocyanates (ITCs)	In vitro (AGS cell line)	0.25 or 0.50 μM	Antiproliferative	Decreased AGS cell invasion and migration	[33]
	In vitro (Human gastric cell lines MKN45, AGS, MKN74 and KATO-III)	3–5 µmol PEITC/g	Chemopreventive effect	-	[52]
Sulforaphane	In vitro (BGC-823 and MGC-803 cell lines)	5 and 10 μM	Apoptosis	Cell cycle arrest and apoptosis induction in GC cells	[34]
	In vitro (Gastric cancer cell lines, BGC-823 and SGC-7901)	0 to 10 μM	Apoptosis	Both BGC-823 and SGC-7901 tumor spheres showed a reduction in the proliferation-related proteins PCNA and Cyclin D1. Bcl-2 expression was reduced in tumor spheres, whereas the expression of the pro-apoptosis proteins Bax and Caspase 8 was elevated	[53]
Alantolactone (ALT)	In vitro (BGC-823 cells)	0, 10, 20, 40 or 60 µM	Apoptosis	Via reduction of AKT signaling, ROS formation was successfully suppressed by the ROS scavenger N-acetyl cysteine, leading to the induction of apoptosis through the action of ALT.	[54]
	In vitro (BGC-823 and SGC-7901 cells)	10 and 20 mM	Anti-proliferation and apoptosis	Gastric cancer cells were killed by alantolactone, perhaps through control of the expression of MMPs.	[35]
Baicalein	In vitro (gastric cancer cell line SGC-7901)	0, 15, 30, and 60 μmol/L	Apoptosis	Revealed Bcl-2 downregulation and Bax overexpression after treatment with baicalein. According to these findings, baicalein causes apoptosis in gastric cancer cells through the mitochondrial mechanism.	[55]
Thymoquinone(TQ)	In vitro (MGC80-3 and SGC-7901)	0–10, 25 µM	Apoptosis	To promote apoptosis, TQ modulates the expression of pro- and anti-apoptotic markers in gastric cancer cell lines. Bacterial Bax and caspase-3 were highly up-regulated whereas Bcl-2 was dramatically decreased.	[56]
Lycopene	In vitro (gastric adenocarcinoma; ATCC CRL 1739)	0.5, 1, or 2 μM	Apoptosis	Increased apoptotic indices (DNA fragmentation, AIF, caspase-3 and caspase-9 cleavage, Bax/Bcl-2 ratio) were seen in the presence of lycopene.	[38]
	In vitro (The gastric epithelial cell line AGS)	25 μg/mL	Anti-proliferative	-	[57]
Alliin and allicin	In vitro (The human gastric adenocarcinoma cell line SGC7901)	15–120 μg/mL	Anti-proliferative	-	[40]
	In vitro (SGC-7901 cells)	30 μg/mL	Apoptosis	-	[40]

### 4.2. Colon Cancer

Colorectal cancer is a major public health concern, as it is one of the leading causes of mortality and illness in the world. With 1.4 million cases in 2012, it was responsible for roughly 9% of all cancer cases [58]. A polyphenolic phytoalexin, resveratrol, which is present in grapes and wine, has been demonstrated to have a number of medicinal qualities. The proliferation of CaCo-2 human colon cancer cells and the metabolism of polyamines were examined in relation to resveratrol’s effects. Resveratrol reduced CaCo-2 cell growth by 70 percent at 25 M. While the molecule arrested the cell cycle at the S/G2 phase, there was no cytotoxicity or apoptosis. Resveratrol inhibited ornithine decarboxylase activity, a key enzyme in the synthesis of polyamines. Reduced putrescine levels in cells after ornithine decarboxylase inhibitor treatment were shown to be linked to resveratrol’s anti-proliferative properties [59]. In a wide range of solid tumor types, notably colorectal cancer, MACC1 (Metastasis Associated in Colon Cancer 1) is a critical regulator and predictive biomarker for colorectal cancer growth and metastasis. MACC1 inhibitors have not yet been found, however. As a result, researchers have used luciferase reporter-based high-throughput screening using the ChemBioNet library of more than 30,000 chemicals to target MACC1 expression. The most effective MACC1 transcriptional inhibitors were found to be the small compounds rottlerin and lovastatin. They dramatically lowered the expression and activity of the MACC1 promoter, which decreased cell motility. Lovastatin reduced the ability of c-Jun and Sp1 to bind to the MACC1 promoter, preventing MACC1 transcription. Most significantly, lovastatin and rottlerin inhibited MACC1 expression and liver metastases in colorectal cancer-xenografted mice. This is the first discovery of inhibitors preventing the spread of cancer and its unique target MACC1. Patients with colorectal cancer may benefit therapeutically from this medication repositioning [60].

Cell death is induced in human colon cancer cells by resveratrol. The pro-apoptotic proteins Bax and Bak relocalize to mitochondria when Caspase is activated. The apoptotic response to the drug is unaffected by inhibition of the Fas/FasL link. Resveratrol induced Fas clustering and redistribution in SW480 cells’ cholesterol and sphingolipid-rich fractions. The death signaling complex is implicated in this redistribution (DISC). FADD, E8, and MC159 viral proteins can be transiently transfected into SW480 cells to lower the apoptotic response and attenuate Bax and Bak conformational changes [61]. Resveratrol inhibited Caco-2 cell growth and proliferation by reducing the incorporation of the dye crystal violet, the radiotracer (3H)-thymidine, and the radiotracer (14C). After being treated with 200 μmol/L of resveratrol for 24 and 48 h, the activity of caspase-3 was considerably increased, resulting in apoptosis. While up to 50 μmol/L had an effect on cell cycle progression, higher doses restored S phase arrest. Only resveratrol had these advantages, while the stilbene analogs stilbenemethanol and rhapontin did not. It was shown by immunoblotting that cyclin D1 and Cdk4 protein levels were decreased. The expression of cyclin E and cyclin A was likewise boosted by resveratrol. Both PCNA and cdk2 proteins were unchanged by the treatment. Resveratrol suppressed the cell cycle in the colon cancer cell line HCT-116 without affecting cyclooxygenase activity. S phase block reversal may be due to hypophosphorylation of the retinoblastoma protein in Caco-2 cells at 200 μmol/L. Cell cycle inhibition in colon cancer cells is reduced by resveratrol [62]. Oxygen demand, mitochondrial biogenesis, and fatty acid oxidation have been shown to be associated with cytotoxic effects of resveratrol. After partial reversal of the Warburg effect, ROS production and mitochondrial membrane hyperpolarization increased apoptosis in cells [63]. Cell growth was inhibited in both lines by resveratrol at a dose-dependent rate. Telomerase activity may be significantly reduced by this drug at larger doses as well. Based on early study results, there is now evidence that resveratrol can be utilized to treat human colon cancer cells [64]. Colon cancer cell proliferation and S phase accumulation are reduced by resveratrol [65]. Treatment with resveratrol resulted in an increase in ER stress markers in human colon cancer cells. In addition, resveratrol elevated GRP-78, which indicates ER stress. Inhibition of caspase-4 activity by z-LEVD-fmk decreased apoptosis produced by resveratrol [66].

Resveratrol can reduce cell development and alter metabolism in colon cancer cells. Increased oxidative capability and decreased glycolysis are the primary effects of resveratrol on the lipidome. The pyruvate dehydrogenase complex is targeted by resveratrol, resulting in an increase in pyruvate dehydrogenation activity. Increases in mitochondrial oxidative capacity and PDH activity that are produced by resveratrol appear to be inhibited by calcium chelation and blockading of the mitochondrial calcium uniport. In addition, inhibiting the CamKKB or downstream AMPK pathway represses the resveratrol-induced increase in glucose oxidation [67]. Even after treatment with IGF-1, resveratrol suppressed HT-29 cell growth by increasing p27 and inhibiting cyclin D1. Resveratrol has been shown to inhibit the growth-regulating Akt/Wnt signaling pathways and IGF-1R protein levels. Different signaling pathways are affected similarly when IGF-1R siRNA is used as a target. Resveratrol induces apoptosis, whereas IGF-1R siRNA does not. Similar to IGF-1R siRNA treatment, resveratrol seems to enhance apoptosis by inhibiting the IGF-1R and its downstream signaling pathways [68]. Resveratrol has been shown to be effective against DLD1 and HCT15 colon cancer cells. Resveratrol induced apoptosis and G1 phase arrest in colon cancer cells, inhibiting cell proliferation. Resveratrol treatment decreased cyclin D1 and E2 protein expression and BCL2 apoptotic regulator BCL2 while enhancing BCL2-associated X and tumor protein p53. AKT1 and AKT2 have been identified as novel resveratrol targets by in silico computational screening. AKT1 and AKT2 have three or four hydrogen bonds in their active pockets, which are thought to be involved in resveratrol’s mechanism of action. Using a pull-down test, it has been found that resveratrol binds to AKT1 and AKT2. Similarly to resveratrol treatment, the deletion of AKT1 and AKT2 in colon cancer cells inhibits cell proliferation and colony growth by delaying cell cycle progression and increasing apoptosis in the cells [69].

Resveratrol has been shown to lower the viability of colon cancer cells. Resveratrol exerted a significant effect on cell viability at 30 μM for at least 48 h. After being exposed to 30 μM resveratrol for 72 h, HCA-17, SW480, and HT29 cells had their viability decreased by 18, 29, and 34%, respectively. Both cancer cell lines died as a result of this treatment. Following only a single 48-h treatment, resveratrol enhanced the proportion of cells that underwent programmed cell death in HCA-17 and SW480 cells by 59% and 67.24%, vs. 2.1% in control cells. Cyclooxygenase-2 and prostaglandin receptor expression in colon cancer cells decreased after treatment with resveratrol. In tests on colon cancer cells, the cyclooxygenase-2 inhibitor indomethacin and cyclooxygenase-2 silencer RNA exhibited similar results [70]. Curcumin is another potential anticancer agent, although its molecular targets remain a mystery. The tumor cellular proteasome is likely to be targeted by curcumin. According to nucleophilic susceptibility and in silico docking studies, the hydroxyl group of the NH2-terminal threonine of the proteasomal chymotrypsin-like component may attack both curcumin’s carbonyl carbons. Curcumin’s CT-like effect is strongly inhibited by purified 20S and 26S proteasomes from rabbits and cells, respectively. Curcumin affects the operation of the proteasome in human colon cancer HCT-116 and SW480 cell lines, resulting in ubiquitination and death of these cell lines. Colon tumor–bearing ICR SCID mice benefited from curcumin, which inhibited the proteasome, decreased tumor growth, and increased tumor apoptosis [71]. Numerous genes involved in the G2/M phase transition were changed by curcumin in HT29 cells. The G2/M cell cycle was stopped by flow cytometry. After 12 and 24 h, curcumin boosted the expression of genes involved in phase II metabolism. Cytochrome P450 genes were reduced in HT29 and Caco-2 cells by curcumin. In addition, curcumin affected metallothionein, tubulin, and other genes involved in colon carcinogenesis [72].

Several human colon cancer cell lines, including CaCo-2 and HT-29, have shown that curcumin affects EGFR gene expression. Researchers investigating Moser cells found an Egr-1 binding site in the egfr promoter, which they believed might be a curcumin response element. Using electrophoretic mobility shift studies, curcumin reduced the DNA binding activity of Egr-1 to the curcumin response element. Additionally, curcumin inhibited Egr-1 transactivation activity by decreasing gene expression, which was achieved by blocking the ERK signal pathway and reducing Elk-1 phosphorylation and activity [73]. Early in colorectal carcinogenesis, the Wnt/-catenin pathway is abnormally regulated. When quercetin was tested on SW480 cells’ -catenin/Tcf signaling, it was shown to be effective. This mode of inhibition was shown to be linked either directly to the -catenin gene itself or to its downstream components in SW480 and HEK293 cells transiently transfected with the constitutively active mutant -catenin gene. Quercetin substantially decreased Tcf complex binding to its specific DNA-binding sites, as shown by EMSA. By immunoprecipitation, quercetin decreased -catenin’s binding to Tcf-4. According to the results of a Western blot test, quercetin decreased the nuclear levels of -catenin and Tcf-4 product [74]. This molecule reduces the development of both benign and malignant cells by activating the cellular energy sensor AMPK. According to this study, Quercetin induces apoptosis in HT-29 colon cancer cells. Quercetin has a dose-dependent effect on cell survival. Within 48 h, quercetin increased G1 cell cycle arrest and apoptosis-related proteins such as AMPK, P53, and P21. Tumor volume was decreased by 50% over six weeks with quercetin treatment, and quercetin-induced apoptosis-related protein synthesis was greater in the 100 mg/kg treated group than in the controls, according to in vivo studies. Quercetin induces apoptosis in HT-29 colon cancer cells through activating AMPK and p53-dependent apoptosis [75]. Quercetin inhibits cell proliferation and induces mortality in colon cancer cells by reducing ErbB2/ErbB3 signaling and activating the Akt signaling pathway [76].

In HCT116 cells, apigenin slowed cell growth in a dose-dependent manner. According to flow cytometry, apigenin prevented cells from entering the G2/M phase. Cell cycle regulators such as p53 and the p53-dependent p21CIP1/WAF1 were similarly decreased by apigenin, while cyclin B1 and its activating partners Cdc2 and Cdc25c were increased. Increased PARP cleavage was accompanied by a decrease in procaspases -8, -9, and -3. Autophagosomes and acidic vesicular organelles accumulated in the apigenin-treated cells, as shown by flow cytometry. Western blot analysis showed that apigenin increased the levels of LC3-II, the processed form of LC3-I. Apoptosis and PARP cleavage were enhanced by 3-methyladenine (3-MA), an autophagy inhibitor. It has been shown that apigenin causes HCT116 colon cancer cells to undergo apoptosis and autophagy. For the treatment of colon cancer, apigenin in combination with an autophagy inhibitor may be useful [77]. At the end of 72 h, 24.92 percent of cells were apoptotic (*p* < 001). A higher percentage (29.13%) was obtained with the same dose of apigenin and 5-FU incubated for the same period of time (*p* < 001). caspase-3 and caspase-8 mRNA and protein expression both increased 2.567-fold and 3.689%, respectively, above control group levels. It was shown, however, that both the expression of the cyclin D1 and the mTOR genes was decreased by an average of 0.423 and 0.231 folds. To our knowledge, this is the first study to show that apigenin alone induces cell cycle arrest and death in HT29 cells. It further demonstrates that apigenin in combination with 5-FU has a stronger effect. This study shows that apigenin may be useful in combination with a cytotoxic chemotherapy medication. A shorter incubation period and lower dose should be used to target the caspase cascade, according to this study [78].

Sulforaphane is a kind of isothiocyanate that, in a dose-dependent way, can stop the cell cycle and kill malignant cells. The increased expression of cyclin A and B1 has been shown to be a contributing factor to this sulforaphane-induced cell cycle arrest. Sulforaphane can trigger apoptosis in cells. Cells treated with phosphatidylserine display plasma membrane phospholipid translocation, chromatin condensation, and ultrastructural alterations associated with apoptosis. While sulforaphane had no effect on p53, elevated levels of proapoptotic Bax protein, mitochondrial release of Cytochrome C, and proteolytic cleavage of Poly(ADP-ribose) polymerase in the test subjects was recorded [79].

Alantolactone improved the oxaliplatin-induced growth inhibition and apoptosis in HCT116 and RKO cells, caused by intracellular ROS accumulation and activation of the JNK and p38 MAPK signaling pathways. HCT116 and RKO cells die as a result of these modifications. ROS reversal medication NAC substantially decreased apoptosis induced by combination therapy and lowered activation of the JNK and p38 pathways in HCT116 and RKO cells. A xenograft model showed that using a combination of drugs outperformed using just one. Tumor growth after treatment was dramatically decreased when compared to either therapy alone [80]. Apoptosis and morphological changes in HCT116, A549, and Panc1 cells were induced by Baicalein, while DEPP, growth arrest, DNA damage-inducible 45 mRNA, and protein levels were enhanced (Gadd45a). MAPK phosphorylation was likewise elevated when DEPP was overexpressed. Baicalein induces apoptosis in HCT116 cells treated with short interfering RNA against DEPP or Gadd45a. DEPP or Gadd45a suppression inhibited the activation of caspase3 and caspase9 and the phosphorylation of MAPKs produced by baicalein. Gadd45a expression was decreased by SP600125/SB203580 inhibition of JNK/p38 activity, although SCH772984 inhibition of extracellular signal-regulated kinase had no effect [81]. Doses of baicalein and wogonin lowered expression of Bcl-2 while increasing expression of Bax. Apoptosis was accompanied by PI3K/Akt inactivation in a dose-dependent manner. HT-29 xenografts in mice were suppressed without damage by baicalein after five weeks of treatment. A possible therapeutic or preventative role for baicalein in the treatment of HT-29 colon cancer cells has therefore been shown [82]. To prevent the formation of COX-2, the thymoquinone treatment reduced p-PI3K, p-Akt, p-GSK3, and beta-catenin levels. EP2 and EP4 activation decreased as a result of decreased COX-2 expression. In LoVo cancer cells, TG treatment reduced the nuclear translocation of -catenin. Additionally, the cofactors LEF-1 and TCF-4 were decreased in the nucleus following thymoquinone treatment. At the transcriptional level, low dose thymoquinone therapy reduced LoVo cell movement by suppressing COX-2 expression. Tumor xenograft studies in nude mice corroborated the effects of PGE2 and thymoquinone on the immune system [83]. At 2 μM, Thymoquinone increased the overall cell death index and triggered apoptosis, although this was reduced with increasing doses. Cell death caused by autophagy was investigated as well. Autophagy was triggered by thymoquinone, which permeabilized mitochondrial outer membranes. The anti-autophagy drugs SP600125 and SB203580 inhibited thymoquinone autophagy by blocking JNK and p38. Prior to autophagy, thymoquinone induced apoptosis, and autophagic cell death was initiated during the creation of autophagosomes. Thymoquinone induced autophagic cell death in CPT-11-R LoVo colon cancer cells by permeabilizing the mitochondrial outer membrane and activating JNK and p38 [84].

Using human colon cancer HT-29 cells, researchers have found that lycopene has an IC50 value of 10 M, indicating that it can effectively inhibit cell growth. Lycopene inhibited Akt activation and decreased the amount of beta-catenin protein that was not phosphorylated in human colon cancer cells. Lycopene has been shown to enhance the phosphorylated form of beta-catenin proteins according to an immunocytochemical study. The promoter activity of cyclin D1 and the synthesis of its protein have both been shown to be decreased as a result of these impacts. Aside from this, lycopene can prevent human colon cancer cells from overproducing the p27kip nuclear cyclin-dependent kinase inhibitor in colon cancer cells, resulting in an increase in its abundance [85]. In human colon cancer HT-29 cells, lycopene exhibited a considerable inhibitory impact on leptin’s capacity to stimulate cell invasion and MMP-7 synthesis. Lycopene has the potential to boost the production of E-cadherin proteins as well as increase their stability [86]. When luteolin was present at a concentration of 100 μM, the expression of non-P-catenin, phosphorylated (inactive) glycogen synthase kinase-3β, and cyclin D1 were all decreased in HCT-15 cells according to Western blot analysis. A flow cytometric analysis revealed that luteolin induced apoptosis in HCT-15 cells as well as a considerable stoppage of the cell cycle in the G2/M phase due to cell cycle arrest in HCT-15 cells. Furthermore, luteolin treatment enhanced the expression of Bax and caspase-3 while decreasing the expression of Bcl-2 according to the findings of a Western blot analysis [87].

The anticancer effect of luteolin has been shown in HCT116 cells by an increase in both p53 phosphorylation and the expression of p53 target genes, resulting in apoptosis and cell cycle arrest. The capacity of luteolin to activate autophagy in p53 wild-type cells but not in p53 mutant cells supports the theory that luteolin’s ability to do so is dependent on p53 function [88]. The quantity of cyclin D1 and c-myc mRNA generated was reduced by caffeic acid phenethyl ester in a dose- and time-dependent manner. In HCT116 and SW480 cells, researchers found that caffeic acid phenethyl ester had a dose-dependent inhibitory influence on the transcriptional activity of beta-catenin/T-cell factor. The quantity of caffeic acid phenethyl ester determined its impact [89]. A cell-cycle analysis of caffeic acid-treated cells demonstrated a higher proportion of cells in the sub-G1 phase. Photomicrographs of the treated cells exhibited blebbing of the cell membrane as well as cell shrinkage. Yo-pro-1 staining of cells treated with caffeic acid suggested apoptosis in a dose- and time-dependent manner. Furthermore, caffeic acid-induced apoptosis was accompanied by an increase in reactive oxygen species (ROS) generation and a reduction in mitochondrial membrane potential [90].

**Figure 4:**
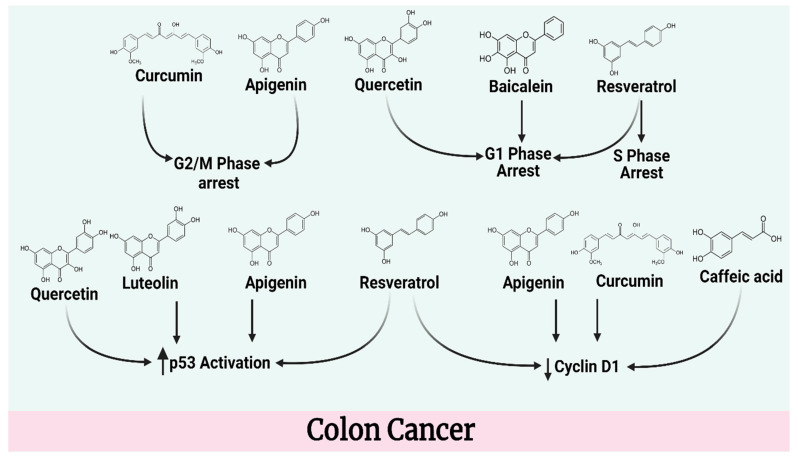
Illustration representing probable sites of blockade by small molecules in colon cancers.

In a variety of human cancer cells, (−)-Epigallocatechin gallate, a physiologically active component of green tea, has been demonstrated to decrease epidermal growth factor receptor activation as well as downstream signaling cascades [91]. Epigallocatechin gallate phosphorylates the stress-inducible kinase p38 mitogen-activated protein kinase (MAPK) and inhibits p38 MAPK via SB203580, a specific p38 MAPK inhibitor, or through gene silencing with p38 MAPK-small interfering RNA (siRNA), which suppresses the internalization and subsequent degradation of EGFR induced by epigallocatechin gallate (Figure 4) [92]. Carotenoids, on the other hand, have been shown to have antiproliferative properties in colon cancer cell lines [93]. Table 2 provides an overview of potential compounds for colon cancer treatment.

### 4.3. Hepatocellular Cancer

Resveratrol has been proven to have neuroprotective, anti-inflammatory, antibacterial, and anti-cancer effects, as well as anti-aging benefits. The molecule has been reported to effectively cause cell cycle arrest and apoptosis, resulting in a significant reduction in Huh-7 cell proliferation. It was able to boost the expression of p21/WAF1 independently of p53 while decreasing the expression of cyclin E, cyclin A, and cyclin-dependent kinase 2. Furthermore, it resulted in an increase in the proportion of pro-apoptotic proteins to anti-apoptotic proteins, which was found to correlate to mitochondrial membrane depolarization and increased caspase activation. Resveratrol had no impact on expression of Fas, Fas ligand, ERK 1/2, or p38, although it did have a negative effect on the expression of phospho-ERK and phospho-p38. Furthermore, resveratrol was revealed to promote an increase in the expression of the autophagy-related proteins Atg5, Atg7, Atg9, and Atg12, resulting in autophagic cell death [94]. Resveratrol produced cell cycle arrest in the G1 and G2/M phases, resulting in considerable slowing of cell growth, a decrease in reactive oxygen species production, and activation of apoptosis. Furthermore, it influenced the NO/NOS system by increasing iNOS and eNOS expression as well as NO activity and synthesis. The suppression of NOS enzymes may diminish its antiproliferative effect [95].

Curcumin administration resulted in a dose-dependent decrease in H22 cell proliferation and an increase in H22 cell death in vitro. Curcumin treatment has been demonstrated to reduce tumor formation in vivo at doses of 50 and 100 mg/kg, respectively. Furthermore, curcumin therapy reduced VEGF expression as well as PI3K/AKT signaling [96]. Tetrahydrocurcumin and curcumin administered together inhibited the expression of hepatocellular carcinoma cells [97]. Quercetin has been shown to be effective in preventing the development of hepatic ellular carcinoma (HCC) in vivo and in vitro. The capacity of quercetin to interfere with the proliferation and cell cycle distribution of LM3 cells may prompt apoptosis. Quercetin increased autophagy in HCC cells while simultaneously suppressing the migration and invasion of LM3 cells. These results were based at least in part on quercetin’s capacity to decrease JAK2 and STAT3 activation, which was confirmed [98]. In vitro studies revealed that quercetin efficiently reduced human HCC cell proliferation and induced apoptosis by upregulating Bad and Bax expression while concurrently downregulating Bcl-2 and Survivin expression. In addition, both in vitro and in vivo studies have shown quercetin to be helpful in suppressing tumor growth and enhancing the therapeutic efficiency of 5-fluorouracil [99].

Apigenin reduced the rate of cell growth and increased the rate of cell death in HepG2 cells in a dose- and time-dependent manner. According to the results of the study, apigenin treatment increased the number of GFP-LC3 puncta as well as the expression of LC3-II. Furthermore, using 3-MA to block autophagy and silence the Atg5 gene increased the apigenin-induced decrease of proliferation and apoptosis. According to these results, apigenin-induced autophagy seems to protect cells against death. Apigenin might potentially block the PI3K/Akt/mTOR pathway, resulting in the activation of apoptosis and autophagy. In vivo results have shown that apigenin injections can suppress tumor formation and that autophagy inhibition by 3-MA can significantly boost apigenin’s anticancer effect. Apigenin inhibits cell growth by suppressing the PI3K/Akt/mTOR pathway, which results in autophagy activation. These effects have been shown in tandem [100]. Apigenin prevented HCC cell invasion and migration, lowered Snail and NF-kB expression, reversed EMT marker increases, enhanced cellular adhesion, controlled actin polymerization and cell migration, and increased cellular adhesion. Apigenin also stopped EMT marker levels from rising. Apigenin may thereby prevent EMT in human HCC by inhibiting the NF-kB/Snail pathway [101]. The angiogenesis-related activities of HUVEC cell survival, migration, and tube formation were all hindered by sulforaphane. Each of these steps is necessary for the formation of new blood vessels. HepG2-stimulated HUVEC migration, adhesion, and tube formation were all significantly inhibited by sulforaphane. The capacity of sulforaphane to disrupt STAT3/HIF-1/VEGF signaling in HepG2 cells may have generated this result. Sulforaphane significantly reduced the growth of HepG2 tumors in a test employing a modified chick embryo chorioallantoic membrane (CAM). This was linked to a decrease in HIF-1 and VEGF production inside the tumors [102]. The expression of telomerase reverse transcriptase is downregulated by sulforaphane, which inhibits cell viability and telomerase activity (hTERT). The researchers proposed that elevated intracellular reactive oxygen species (ROS) levels induced by sulforaphane exposure might play a substantial role in the abolition of hTERT expression, as pretreatment with NAC, an antioxidant, resulted in the recovery of hTERT expression. The ability of sulforaphane to diminish the phosphorylation of Akt at Ser-473 suppressed hTERT phosphorylation. This impact was reversed by using NAC prior to sulforaphane exposure [103].

Baicalein has been used in traditional Chinese medicine for its anti-inflammatory and anti-cancer qualities for generations. Treatment with baicalein reduced the production of matrix metalloproteinase-2 (MMP-2), matrix metalloproteinase-9, and urokinase-type plasminogen activator (u-PA), as well as proteinase activity, in hepatocellular carcinoma MHCC97H cells. Meanwhile, tissue inhibitor of metalloproteinase-1 (TIMP-1) and TIMP-2 expression increased in a dose-dependent manner. Furthermore, baicalein administration resulted in a considerable decrease in the levels of phosphorylated forms of MEK1 and ERK1/2. An increase in MEK1 expression hindered the anti-metastatic effects of baicalein to an extent [104]. There was a synergistic reduction in the expression of MMP-2, MMP-9, and u-PA, as well as an increase in the expression of TIMP-1 and TIMP-2, when MHCC97H cells were treated with an ERK inhibitor (U0126) and baicalein at the same time. Furthermore, the ability of MHCC97H cells to invade was decreased [105]. By causing cell cycle arrest in the S and G2/M phases of the cell cycle, Baicalein was able to reduce the proliferation of Bel-7402 cells. This was achieved by upregulating p21/CDKN1A and P27/CDKN1B expression while downregulating the PI3K/Akt pathway. Baicalein has the ability to alter miRNA expression patterns in Bel-7402 cells [106]. In terms of cell proliferation regulation and cell cycle arrest, prospective target genes for differentially expressed miRNAs might be selected. The MAPK, PI3K-Akt, Wnt, Hippo, and mTOR signaling pathways have all been implicated in these probable target genes. In numerous HCC cell lines, one of the up-regulated miRNAs, miR-3127-5p, has a low expression level. Overexpression of miR-3127-5p, on the other hand, may stop cell development in Bel-7402 and Hep3B cell lines by causing S phase arrest. This is exacted by upregulating p21 and P27 expression while downregulating the PI3K/Akt pathway [107]. Apoptosis is elevated as a consequence of thymoquinone’s elevation of TRAIL/TRAILR2, as shown by the compound’s activation of caspase-3 and downregulation of Bcl-2. Thymoquinone inhibits the development of HCC via three mechanisms: reduced oxidative stress, suppression of TGF-1, and stimulation of TRAIL-mediated apoptosis. Researchers have discovered that thymoquinone dramatically decreases the survival and proliferation of HepG2 cells in a dose-dependent manner. The potential for thymoquinone to initiate apoptosis was determined using flow cytometry and colorimetric assays of Caspases 3 and 9. The apoptotic effect of thymoquinone was significantly more evident after 12 h of treatment, and both caspases-3 and -9 activity was increased. Furthermore, according to flow cytometric analysis of the cell cycle, the cells underwent early G1/S arrest, which is a hallmark of apoptosis [108].

Pretreatment with lycopene enhanced the studied pathways that had been harmed by NDEA in mice that had previously been administered the medication [109]. Furthermore, hepatic electron micrographs from the Lycopene + NDEA group exhibited an increase in macrophages, apoptotic bodies, and well-differentiated hepatocellular carcinoma (HCC), as opposed to the undifferentiated HCC reported in the NDEA-treated group [110]. The effectiveness of luteolin in preventing N-nitrosodiethylamine (DEN)-induced head and neck cancer was studied in albino rats. In the group that was treated with DEN alone, it was discovered that the tissue-damaging enzymes were at a high level, while enzymatic antioxidants were reduced in a harmful manner. In the group that had been treated with the toxin (DEN alone), severe lesions and cirrhosis were detected. The luteolin-treated DEN group caused changes in both the enzymes that damaged tissue and the antioxidants produced by enzymes. The DEN treatment nearly fully repaired the damaged lesion seen in the histoarchitecture of the rat liver. In conclusion, the results of this investigation provide compelling evidence that luteolin has a powerful therapeutic capacity against HCC in albino rats [111].

**Figure 5:**
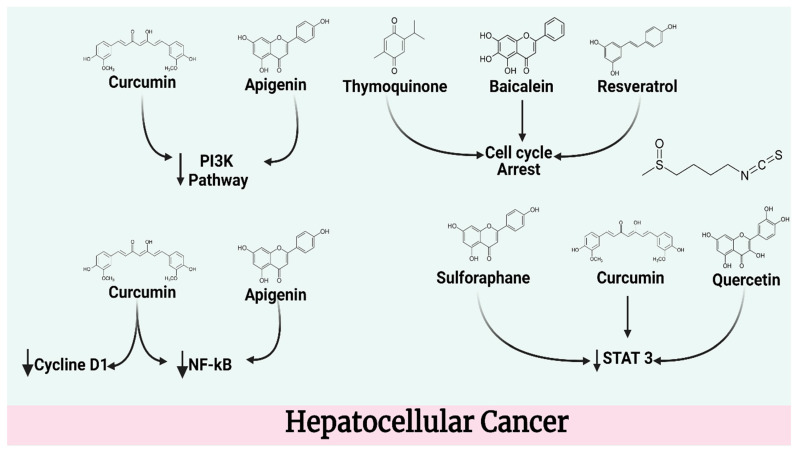
Illustration representing the probable sites of blockade by small molecules in hepatocellular cancer.

Caffeic acid reduced angiogenesis and the CoCl2-induced autocrine synthesis of vascular endothelial growth factor (VEGF) in HCC cells. The expression of HIF-1alpha, as well as phosphorylated signal transducers and transcription-3 activators, increased after CoCl2 treatment, conferring information about cellular processes (p-STAT-3). Then, by connecting to the promoter of the VEGF gene and forming a binding site there, HIF-1 effectively activated VEGF. Caffeic acid, on the other hand, was able to block CoCl2-induced HIF-1 activation, most likely by reducing JNK1 activation and decreasing the quantity of HIF-1 that was stabilized. Furthermore, Caffeic acid was able to attenuate the CoCl2-induced increase in p-STAT-3 expression (Figure 4). The combination of these two activities resulted in a decrease in HIF-1 recruitment to the VEGF promoter [112]. Due to the microRNA-124, HCC cells were able to block the synthesis and release of endogenous IL-6 when caffeic acid was introduced to the (miR-124)-mediated NF-kB-IL-6-STAT-3 feedback loop decrease [113]. The quantity of VEGFR-2 and p-VEGFR-2 proteins expressed in the cells was reduced in a time and dose-dependent manner by epigallocatechin gallate. HuH7 cells produced less of the growth factor VEGF after being treated with epigallocatechin gallate. Consumption of epigallocatechin gallate reduced the growth of HuH7 xenografts in nude mice significantly (Figure 5). This was accompanied by a decrease of VEGFR-2 activation and associated downstream signaling pathways such as ERK and Akt. The expression of Bcl-xL protein and VEGF mRNA in the xenografts was reduced when epigallocatechin gallate was consumed [114]. Table 3 illustrated the overview of potential compounds for hepatocellular carcinoma.

### 4.4. Pancreatic Cancer

The cytotoxic and metabolic effects of N-hydroxy-N′-(3,4,5-trimethoxyphenyl)-3,4,5-trimethoxy-benzamidine (KITC), a newly synthesized polymethoxylated resveratrol analogue, were examined in two human pancreatic cancer cell lines. N-hydroxy-N′-(3,4,5-trimethoxphenyl)-3,4,5-trimethoxphenyl)-3,4,5-trimethoxphenyl)-3,4,5-trime, when compared to a control (KITC) at a concentration of 40 μM, was able to halt cell proliferation in the G0/G1 phase of the cell cycle and lower the number of cells in the S phase by around 105 and 35 percent, respectively. KITC has been demonstrated to significantly reduce the in situ activity of ribonucleotide reductase, an important enzyme in the DNA synthesis process. This decrease was shown to be dose-dependent in both pancreatic cancer cell lines. When growth inhibition studies were carried out, KITC was shown to have a synergistic impact with gemcitabine in both cell lines [116]. Resveratrol decreased the proliferation of four human PaCa cell lines, synergized with the apoptotic effects of gemcitabine, blocked the constitutive activation of NF-kB, and suppressed the expression of bcl-2, bcl-xL, COX-2, cyclin D1, MMP-9, and VEGF. In addition, resveratrol was discovered to enhance gemcitabine’s apoptotic effects [117].

Curcumin was shown to prevent the constitutive synthesis of the cytokine IL-8 in a dose-dependent manner at dosages ranging from 10 to 100 μM. Curcumin treatment resulted in a significant decrease in NF-kB activation. When cancer cells were pretreated with curcumin, their rate of growth was considerably decreased. The addition of exogenous recombinant IL-8 did not alter the curcumin-induced decrease in cell growth. Curcumin greatly increased the expression of both CXCR1 and CXCR2, according to the results of an investigation into the expression of the IL-8 receptors known as CXCR1 and CXCR2. Exogenous IL-8 was unable to restore this increase in IL-8 receptors. According to these studies, curcumin inhibits IL-8 from causing receptor internalization, which enhances pancreatic cancer survival [118].

Quercetin has been revealed to have a cytotoxic effect on cells obtained from both neoplastic cell lines in a concentration-dependent manner. In case of the EPP85-181RDB cell line, it appears that quercetin may render resistant cells more susceptible to daunorubicin. Both drugs had an effect on the sensitive cell line, and the effect was synergistic, meaning that it was better than either chemical acting alone. According to these results, quercetin has the capacity to overcome cancer cells’ resistance to chemotherapeutic effects. In the presence of sensitive tumor cells, however, flavanol has been demonstrated to improve the action of cytostatic medicines. This discovery shows that flavanol may allow cytostatic medication doses to be reduced, lessening therapeutic side effects [119]. By decreasing RAGE expression in pancreatic cancer cells, quercetin has been shown to boost sensitivity to GEM treatment. Quercetin was able to influence apoptosis and autophagy-related pathways via this molecular mechanism [120].

Apigenin has been shown to be able to limit the development of the human pancreatic cancer cell line BxPC-3 in a dose-dependent manner and bring about G2/M phase cell cycle arrest. The results of an immunohistochemistry experiment demonstrated that apigenin concentration increased while Bcl-2 gene expression fell significantly. There was an increase in the expression of the Bax gene at this time as well [121]. Apigenin inhibited DNA synthesis and cell proliferation in four distinct pancreatic cancer cell lines, with the amount of the molecule and the passage of time playing a role, and produced cell cycle arrest in the G2/M phase. Apigenin reduced the levels of cyclin A and cyclin B, as well as phosphorylated forms of cdc2 and cdc25, all of which are required for the transition from G2 to M [122].

Sulforaphane blocked NF-kB binding, downregulated apoptosis inhibitors, and induced apoptosis in the cells, in addition to inhibiting clonogenicity [123]. By orthotopically implanting primary pancreatic CSCs derived from human pancreatic tumors into the pancreas of NOD/SCID/IL2Rgamma mice and treating them with sulforaphane, tumor development was significantly reduced. The regulation of Sonic hedgehog–GLI signaling was responsible for the tumor growth decrease. Inhibition of the hedgehog pathway by sulforaphane at a dose of 20 mg/kg resulted in a 45 percent reduction in the growth of pancreatic cancer tumors in mice, as well as a reduction in the expression of Shh pathway components such as Smo, Gli 1, and Gli 2 in mouse tissues. Sulforaphane has been demonstrated to decrease the expression of pluripotency-preserving transcription factors such as Nanog and Oct-4, as well as angiogenic markers such as VEGF and PDGFRa, which are all downstream targets of Gli transcription. Furthermore, sulforaphane therapy resulted in a significant drop in EMT markers Zeb-1, which correlates with an increase in E-Cadherin expression. This shows that the treatment can inhibit a signaling mechanism linked to early metastasis. It is worth noting that sulforaphane was able to enhance apoptosis by reducing Bcl-2 and XIAP expression. According to these data, inhibition of the Shh system by sulforaphane resulted in a significant drop in EMT, metastatic, and angiogenic markers, as well as a significant reduction in tumor formation in mice. By concentrating on cancer stem cells, therapeutics that target the Shh pathway might be able to improve the prognosis of patients with pancreatic cancer (CSCs). This is because aberrant Shh signaling occurs throughout the pancreatic carcinogenesis process [124].

Alantolactone, a naturally occurring sesquiterpene lactone, significantly inhibited human pancreatic cancer cells and dampened the action of constitutively activated STAT3 [125]. Because autophagic degradation was impaired, alantolactone caused an increase in autophagosomes. In addition, it reduced the activity and expression of CTSB and CTSD proteins, which induce lysosomal dysfunction when depleted. Furthermore, the researchers observed that alantolactone inhibited pancreatic cancer cell development both in vitro and in vivo, as well as increasing pancreatic cancer cell chemosensitivity to oxaliplatin. Furthermore, a drop in TFEB levels is an important step in the alantolactone-induced apoptosis and cell death pathway [126]. Thymoquinone has been shown to exhibit anti-neoplastic and anti-metastatic effects in nude mice with human pancreatic cancer. The expressions of XIAP and MMP-9 may have been downregulated as well [127]. PANC-1 cells treated with lycopene showed lower levels of intracellular and mitochondrial ROS, mitochondrial function (measured by mitochondrial membrane potential and oxygen consumption rate), NF-kB activity, and expression of NF-kB-dependent survival genes. Lycopene treatment reduced cell viability in PANC-1 cells, as shown by increases in active caspase-3 and the ratio of Bax to Bcl-2. Based on these findings, it seems that consuming lycopene as a supplement may help to reduce incidence of pancreatic cancer [128].

The levels of caspase-3 and cleaved PARP increased after luteolin administration for 24 h, and the expression of the pro-apoptotic Bax protein increased while the expression of the anti-apoptotic protein Bcl-2 decreased. This was accompanied by an increase in caspase-3 levels. In vivo, luteolin inhibited the proliferation of HUVEC and the formation of vasculature in CAM. Furthermore, the presence of luteolin resulted in a decrease in the quantity of VEGF in conditioned medium produced from human pancreatic cancer cells. Using a co-culture system, it was shown that pancreatic cancer cells pretreated with luteolin were able to prevent HUVEC from forming capillary-like structures. The inhibition of VEGF mRNA expression and the reduction in VEGF secretion were shown to be linked. An impairment in the transcriptional activity of the nuclear transcription factor might have caused the inhibition of VEGF mRNA expression. NF-kB [129].

**Figure 6:**
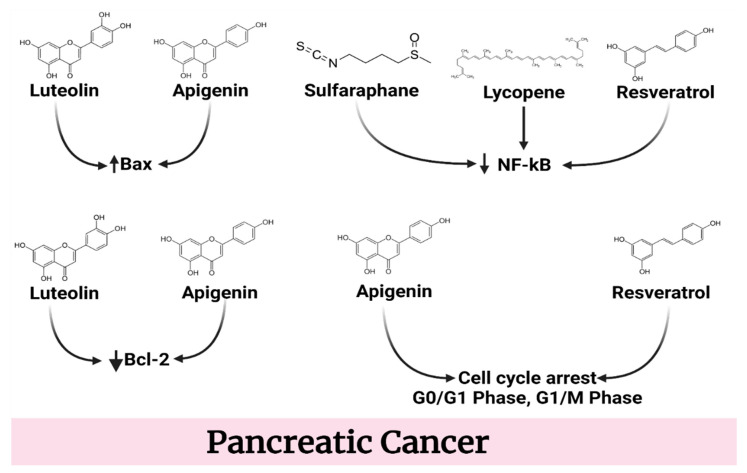
Illustration representing the probable sites of blockade by small molecules in pancreatic cancer.

Caffeic acid is a strong substance that causes cells to die. Side effects of its activities include mitochondrial dysfunction and caspase-3 and caspase-7 activation (Figure 6) [130]. Caffeic acid has the ability to block the orthotopic development and EMT of PANC-1 pancreatic cancer cells, which is followed by a reduction in vimentin and Twist 2 expression [131]. Table 4 provides an overview of potential compounds for pancreatic cancer treatment.

## 5. Synergistic Effects

Colorectal cancer and stomach cancer are the most common types of malignancies affecting the gastrointestinal tract [133,134]. Single-agent and combination epigenetic therapies for gastric cancer have both been examined preclinically. Gastric cancer cell line Somatostatin, which may contain powerful anticancer action, has its promoter DNA methylation increased by AGS; treatment with DAC demonstrates promoter dimethylation and reabsorption of somatostatin [135]. AGS increased the methylation of somatostatin’s promoter [136]. Urine metabolism, which involves a group of amino acids, has been shown to be abnormally high in patients with gastric cancer compared to healthy controls in research on the genesis of gastric cancer [137,138]. The levels of amino acids such as alanine, glycine, valine, serine, isoleucine, thrombin, proline, methionine, tyrosine, and tryptophan were lower in healthy controls [139]. Thrombin, serine, and alanine all had values under the diagnostic curve that were more than 0.8, indicating strong diagnostic value for detecting stomach cancer [140,141]. Furthermore, when paired with a specific HDAC inhibitor, certain amino acids have been demonstrated to precisely predict the potential of trichostatin, resulting in a synergistic increase in somatostatin mRNA expression [135]. This degree of intensity is common in Asian countries such as China, Japan, and South Korea. Because there is no sensitive or exact diagnostic test for stomach cancer, the great majority of patients are discovered after their illness has progressed to an advanced stage and the window for surgical therapy has passed. Chemotherapy approaches such as platinum-based fluorouracil and second-tier treatments such as docetaxel, paclitaxel, or arenotecan may be used to treat patients with advanced astrocytic carcinoma [135]. These therapies are suggested as first-line therapy. While chemotherapy drugs are excellent at killing cancer cells, they can contribute to the development treatment resistance [135]. The anticancer drug 5-fluorouracil (5FU) is effective against the great majority of gastrointestinal malignancies. In the realm of tumor chemotherapy, the utilization of therapeutic medications in combination is presently an active and exciting area of research [135], offering an approach that has the potential to effectively overcome drug resistance. The use of anti-tumor drugs in combination with other treatment modalities may improve the effectiveness of these therapies, reduce the required dosage, provide more effective and better options for the clinical treatment of tumors, and reduce patient suffering [142,143]. Recent pharmacological research has shown that the medicinal plant *Cornus officinalis* possesses immunomodulatory and anti-tumor effects [144,145]. The natural ingredient loganetin is derived from this plant [135]. In related research, the gastric cancer cell lines MGC803 and HGC27 were kept at the Shanghai Institute of Digestive Surgery. Both cell lines were grown in RPMI-1640 medium with 10% fetal bovine serum, 5% carbon dioxide, and 5 micrograms per milliliter of penicillin and streptomycin in a humid incubator at 37 degrees Celsius. RPMI-1640 medium supplement was created for testing the 1% fetal bovine serum and stored at 4 degrees Celsius [146]. The researchers observed that the combination of 5FU and loganetin significantly reduced cell efficiency when compared to the medication administered alone [135]. In order to learn more about whether these treatments have a synergistic impact on cancer cells, researchers produced combination index (CI) values in the range of 5FU and loganetin dosages. [135]. Synergy is consistent with a CI value of less than 0.90. In their study, they observed that the CI values in the gastric cancer cell line were both smaller than 0.90, indicating that 5FU and lignin had a synergistic effect. The ideal concentration for different animals was calculated based on the association between stomach cancer cell activity and CI values [135].

## 6. Clinical Evidences

According to clinical and epidemiological investigations, chronic inflammation is associated with a range of gastrointestinal cancers. People with autoimmune disorders such chronic hepatitis B, *Helicobacter pylori* infection, or inflammatory bowel disease, for example, are more likely to develop liver cancer, gastric cancer, or colorectal cancer, respectively [135]. Furthermore, solid tumors share many common characteristics with swollen tissues, a phenomenon known as tumor-induced inflammation. The presence of proinflammatory mediators, including cytokines, chemokines, and lipids, as well as infiltration of poorly controlled immune cells and the presence of endothelial cells and fibroblasts, are all typical clinical characteristics. Chronic inflammatory disorders and cancers are both frequent [135]. The fact that nonsteroidal anti-inflammatory medicines diminish the incidence, metastasis, and mortality of numerous solid tumors backs up the theory that chronic inflammation plays a role in the development, growth, and progression of cancer [135]. The digestive system can be affected by cancer, in various forms; after DNA damage, the processes of cell division, proliferation, and growth are all regulated by the cell cycle, which is an important regulator of these activities [147]. This method is often divided into four separate parts, depending on the situation; G1 and G2 are two unique length gaps used to differentiate between the S phase (where DNA is produced) and the M phase (where mitosis occurs), respectively [135]. Apoptosis is induced when several chemotherapeutic drugs fail to exert their intended effect on the treated cells. On the other hand, apoptosis and the cell cycle are intricately linked, as shown by the essential role that p53 plays in inhibiting both the cell cycle and apoptosis. Apoptosis and the cell cycle are closely linked. In cases where the cell cycle is disrupted, p53 activation protects DNA integrity [135]. The human colon cancer cell line HCT116, with the exception of CDKI p21, serves as a prime example of the role that the cell cycle plays in reducing the effectiveness of chemotherapy. Because HCT116 has effectively demonstrated its effectiveness, it is widely employed [135]. A normal p21 (HCTp21+/+) cell, when irradiated by gamma-radiation, inhibits the growth phase of the cell cycle, after which the clonogenic cell survives. Where p21 (HCTp21−/−) is deficient, when irradiated by gamma-radiation, the cell cycle does not pass through growth inhibition and progresses to apoptosis. Clonogenicity is an result of a newly targeted drug called flavopyridol, which has the ability to induce a cell to move from a state where it is stuck in the cell cycle to a state where it commits suicide [148]. On the other hand, it depends on the presence of single cells containing wild-type p53 in addition to the intact p53 and p21 axes [149]. Despite this requirement, about 50% of all gastrointestinal cancers contain a mutation in p53, and even cells with the mutant p53 can enter the G2 cell cycle when treated with DNA-damaging drugs such as CPT-11. As a result, the development of an alternative approach to the treatment of cancer is a problem that needs to be addressed. When examining the cell cycle, it is possible to gain new insights into the many ways in which a cell can be modified. One of these mechanisms is the function that converts mutant p53 from cell cycle arrest to cell death [150,151]. To investigate the expression of PD-L1 in clinical settings, immunohistochemistry staining (IHC) tests for PD-L1 are often performed using pretreatment tissue samples obtained by biopsy. These studies are usually carried out on tissue samples. These samples come from the primary tumor that was found in the patient. Nevertheless, several studies have shown that certain clinical situations may favor an accurate assessment of a person’s PD-L1 levels. These problems are probably related to the importance of the expression of PD-L1 as a predictive biomarker for PD-1/PD-L1 inhibition. Researchers have examined the technical and biological problems with PD-L1 evaluation from a clinical point of view [135].

## 7. Concluding Remarks and Future Directions

Many clinical trials have studied the effects of targeted treatment on gastric cancer, although its use in stomach cancer is greater than its effective use in children with colon, lung, and breast cancer. As natural small molecules lack selectivity towards these receptors, it is necessary to modify the structure in order to enhance selectivity. A targeted stomach cancer therapy faces several challenges. While studies have been carried out, few clinical trials have been conducted. A targeted therapy for stomach cancer is only considered to be effective if a third-stage clinical trial is completed. The pathophysiology of gastric cancer is highly complex, and most drugs have thus been used for treatment in only a few cases. There are several factors that contribute to gastric cancer drug trial failure. Multiple-target pharmaceuticals or targeted therapeutics can replace cancer therapy when used in combination with innovative surgical, radiological, and chemotherapeutic procedures. Novel therapeutic approaches may not work for every patient because of unique individual factors, an important input when considering biomarkers and related genes for making specific drugs. Drugs is expected to be widely available in clinical settings. The molecular process of tumor growth can lead to efficient personal therapy for gastric cancer despite these obstacles, which should usher in a new chapter in the fight against advanced gastric cancer. The success rate in treating gastric cancer patients is low because of this cancer’s unique biological properties and internal and acquired drug resistance mechanisms. Advances in genetics and molecular biology have led to the development of innovative therapeutic options for stomach cancer. To combat gastric cancer, researchers have discovered potential anti-cancer therapy goals that are involved in the progression and spread of the disease. Flavopyridol (Flavopyridol inhibitor drug) and cell cytoplasm are newly discovered pharmaceuticals that target receptor/receptor kinase inhibitors (such as Flavopyridol, Flavopyridol inhibitor drug) and cytoplasm. “Fancy” or “personal” drugs may increase cancer cell death, apoptosis, chemosensitivity, or growth inhibition for their specific biological purposes. People with HER2-positive gastric cancer are currently being evaluated in randomized trials using trastuzumab (ToGA). In a clinical trial, 22.1 percent of patients with HER2-positive malignancy were treated with 5-FU with or without capsitabine and CDDP trastuzumab. Compared to patients receiving chemotherapy alone, patients treated with trastuzumab lived an average of 13.5 months longer than those who did not receive the medication. The treatment of gastric cancer can benefit from this research, which has already created a wave of research into a new combination of chemotherapy and molecular targeted therapy.

## Figures and Tables

**Table 2:** Natural small molecules in colon cancer.

Name of Compound	Subject	Dose	Result	Mechanism of Action	Reference
Resveratrol	In vitro (CaCo-2 human colon cancer cells)	25 μM	70% growth inhibition	Reduction in the amount of ornithine decarboxylase enzyme	[59]
	In vitro (SW480, SW620, and HCT116 cell line)	10–100 μm	Cell death	Conformational changes of Bax and caspase activation	[61]
	In vitro (Caco-2 cell)	12.5–200 μmol/L	Inhibition of cell development and proliferation	Increased activity of caspase-3, S-phase arrest, reduced production of cdk-4 and cyclin D1	[62]
	In vitro (SW620 cell)	10 μM	Cell growth inhibition	Elevation of mitochondrial oxygen consumption rate and ATP production	[63]
	In vitro (WiDr and HT-29 cell)	5, 10, 20 and 40 µg/mL	Reduction in cell viability	Inhibition of TLMA and reduced activity of telomerase	[64]
	In vitro (SW620 and SW480)	30 µM	Cell proliferation inhibited	S-phase arrest, elevated initiation of cyclins B and A expression, increased level of Cdk 2 protein, increase in phosphorylated histone H2AX, activation of p53 protein, activation of ATR/p53 pathway	[65]
	In vitro (HT-29 cell)	50 and 100 µM	Reduction in cell progression	Endoplasmic reticulum stress, inhibition of caspase-4	[66]
	In vitro (Caco2 colon cell)	10 µM	Reduction in cell progression	Inhibition of pentose phosphate pathway, S phase arrest	[67]
	In vitro (SW480 and HT-29 cells)	100–150 μM	Reduction of cell proliferation	P27 stimulation, cyclin D1 suppression, IGF-1R suppression, p53 activation	[68]
	In vitro (HCT15 and DLD1 cells)	10, 20, 30, and 40 μM	Reduction of cell proliferation	G1 phase arrest, reduced cyclin D1, E2, and BCL2 expression, elevated P53 level.	[69]
	In vitro (SW480 and HT-29 cells)	30 μM	Cell death	Inhibition of COX-2 and reduced expression of PEG2	[70]
Curcumin	In vitro (SW480 and HCT116 cells)	10, 20, and 30 μmol/L	Cell proliferation inhibition	Inhibition of 20S proteasome activity	[71]
	In vitro (HT-29 cells)	-	Reduction in cancer cell	G2/M cell cycle arrest, downregulation of cytochrome P450 gene	[72]
	In vitro (Moser cell)	0, 5, 10, 15 and 20 μM	Cell progression inhibition	Reduced expression of EGFR and cyclin D1	[73]
Quercetin	In vitro (HEK293 and SW480 cells)	0–100 μM	Cell progression inhibition	Inhibition of Beta-catenin/Tcf signaling pathway	[74]
	In vitro (HT-29 cells)	100 mg/kg	Inhibition of cancer cell development	G1 cell cycle arrest, upregulation of p21, AMPK, and p53	[75]
	In vitro (HT-29 cells)	0, 25, 50, and 100 μmol/L	Apoptosis and cell viability decline	Restriction of ErbB2/ErbB3 signaling, Akt pathway, and Bcl-2 level	[76]
Apigenin	In vitro (HCT116 cells)	25 and 50 μM	Apoptosis induction	G2/M phase cell cycle inhibition, suppression of cyclin B1, elevated expression of P53 and p53-dependent p21CIP1/WAF1, decreased level of procaspase-8,9,3	[77]
	In vitro (HT29 cell)	90 μM	Induced apoptosis	Caspase-3 and caspase-8 expression increased, mTOR and cyclin D1 expression decreased	[78]
Sulforaphane	In vitro (HT29 cells)	5–50 μM	Induced apoptosis	Increased initiation of cyclin A and cyclin B1	[79]
Alantolactone	In vitro and In vivo (HCT116, RKO cells and BALB/c mice)	40–120 µM	Cancer cell death	Activation of JNK and p38 MAPK signaling pathway	[80]
Baicalein	In vitro (panc-1, HTC116 and A549 cells)	0, 10, 20 or 40 µM	Apoptosis	Upregulation of Gadd45a and DEPP	[81]
	In vitro (HT29 cells)	100 μM	Cell viability reduced	G1 cell cycle arrest, reduced Bcl-2 expression, increased Bax expression, and PI3K/AKT pathway inactivation	[82]
Thymoquinone	In vitro (LoVo cells)	20 μmol/L	Inhibited migration and cell growth	p-PI3K, p-Akt, p-GSK3, and beta-catenin levels were all reduced.	[83]
	In vitro (LoVo cells)	2 μM	Cell death	Mitochondrial outer membrane permeability increased when JNK and p38 were activated.	[84]
Lycopene	In vitro (HT-29 cells)	10 μM	Cell proliferation halted	Nonphosphorylated beta-catenin protein and reduced Akt activation	[85]
	In vitro (HT-29 cells)	250 nM	Inhibition of cancer cell progression	Inhibition of MMP-7	[86]
Luteolin	In vitro (HCT-15 cells)	100 µM	Cell cycle arrest and apoptosis	Reduced expression of non-P-beta-catenin, phosphorylated glycogen synthase kinase-3beta, and cyclin D1.	[87]
	In vitro (HT-29 cells)	10–20 μM	Cell cycle blockade and apoptosis	Increased p53 phosphorylation and p53 target gene expression	[88]
Caffeic acid	In vitro (HCT116 and SW480 cells)	2.5, 5 or 10 mg/mL	Apoptosis and cell growth inhibition	Cyclin D1 and c-myc expression were reduced in a dose-dependent manner.	[89]
	In vitro (HCT 15 cells)	800 μM	Induced apoptosis	Increased generation of ROS and decreased mitochondrial membrane potential	[90]
Epigallocatechin gallate	In vitro (HT-29 cells)	5 μg/mL	Inhibits cancer cell growth	Inhibited epidermal growth factor receptor	[91]
	In vitro (SW480 cells)	25 μM	EGFR is downregulated	Activation of p38 MAPK resulted in phosphorylation of EGFR at serine 1046/1047	[92]
Carotenoids	In vitro (HCT116 cell)	-	Cell growth inhibited	Induced apoptosis	[93]

**Table 3:** Natural small molecules in hepatocellular cancer.

Name of Compound	Subject	Dose	Result	Mechanism of Action	Reference
Resveratrol	In vitro (Huh-7 cells)	22.4 μg/mL	Cell cycle arrest and apoptosis induction	Although cyclin E, cyclin A, and cyclin-dependent kinase 2 expression was downregulated, p21/WAF1 expression was elevated in a p53-independent manner	[94]
	In vitro (HepG2 cell lines)	10^−7^ M	Cell progression inhibited	Cell cycle inhibited in G1 and G2/M phase	[95]
Curcumin	In vitro and in vivo (H22 HCC cells and mice)	50 and 100 mg/kg	Cell proliferation inhibited	The signaling pathways PI3K/AKT and vascular endothelial growth factor were inhibited.	[96]
	In vitro (HepG2 cells)	300–3000 mg/kg	Cytotoxic activity	Suppression of NF-kB, AP-1 STAT3, STAT4, peroxisome proliferators-associated receptor gamma, cyclin D1	[97]
Quercetin	In vitro and in vivo (LM3 cell line and mice)	20–200 μmol/L	Induction of apoptosis	Down-regulation of JAK2 and STAT3	[98]
	In vitro (HepG2 and SMCC cell line)	0.05, 0.10, or 0.15 mM	Cell proliferation inhibition and apoptosis induction	Increased expression of Bad and Bax, with a reduction in Bcl-2 expression	[99]
Apigenin	In vitro (HepG2 cells)	10, 20 and 40 μM	Apoptosis and autophagy induction	Eradication of PI3K/Akt/mTOR pathway	[100]
	In vitro (HCC cell lines)	10 and 20 μM	Inhibited the migration and metastasis	Snai1 and NF-kB expression were reduced, but EMT marker levels rose.	[101]
Sulforaphane	In vitro and in vivo (HCC cell line, HepG2 and human endothelial cell)	1.25, 2.5, 5, 10 and 20 μM	Reduced tumor growth	STAT3/HIF-1α/VEGF signaling inhibition	[102]
	In vitro (Hep3B cells)	20 μM	Cell viability inhibited	Downregulation of telomerase reverse transcriptase and suppression of phosphorylation of Akt	[103]
Baicalein	In vitro and in vivo (HCC cell lines and mice)	50 μM	Reduction in cell motility and migration	MMP-2, MMP-9, and u-PA levels fell, while MEK1 and ERK 1/2 were phosphorylated.	[105]
	In vitro (HCC cell line, Bel-7402 and Hep3B)	40 and 80 μM	Cell proliferation inhibited	During the S and G2/M phases, cell cycle arrest was accomplished by upregulating the expression of p21/CDKN1A and P27/CDKN1B and blocking the PI3K/Akt pathway	[107]
Thymoquinone	In vivo (male Sprague rats)	20 mg/kg	Inhibited cancer cell progression	Upregulation of TRAIL/TRAILR2, caspase-3, and Bcl-2	[115]
	In vitro (HepG2)	25, 50, 100, 200 and 400 μM	Inhibition of cancer cell growth	Increase in Caspase 2 and 9; G1/S cell cycle arrest	[108]
Lycopene	In vivo (Female BALB/c mice)	5 mg/kg	Induction of apoptosis	Enhanced expression of PCNA and cyclin D1	[110]
Luteolin	In vivo (Albino mice)	0.2 mg/kg	Induction of apoptosis	Altered tissue-damaging enzymes and enzymatic antioxidants.	[111]
Caffeic acid	In vitro and in vivo (HepG2, HUVECs and BALB/c mice)	20 μM	Attenuation of the angiogenic function	JNK-1-mediated HIF-1α stabilization was reduced..	[112]
	In vitro (HepG2 and MHCC97H)	10, 20 or 40 μM	Inhibits cancer cell progression	Endogenous Interleukin-6 expression was inhibited.	[113]
Epigallocatechin gallate	In vitro (HLF, PLC/PRF/5, HepG2, HuH7, HLE, and Hep3B)	25 µg/mL	Inhibition of cancer cell growth	VEGFR-2 and p-VEGFR-2 protein expression reduced; ERK and Akt signaling pathways inhibited.	[114]

**Table 4:** Natural small molecules in pancreatic cancer treatment.

Name of Compound	Subject	Dose	Result	Mechanism of Action	Reference
Resveratrol	In vitro (AsPC-1 and BxPC-3)	10, 20 and 30 μM	Induction of apoptosis	Arrested cells in the G0/G1 phase of the cell cycle 2 and depleted cells in the S phase	[116]
	In vitro and in vivo (AsPC-1 and Male athymic nu/nu mice)	10 μM	Cell proliferation inhibited	NF-kB activation, as well as the expression of bcl-2, bcl-xL, COX-2, cyclin D1, MMP-9, and VEGF, were all inhibited.	[117]
Curcumin	In vitro (SUIT-2)	10–100 μM	Cell proliferation inhibited	Reduces IL-8	[118]
Quercetin	In vitro (EPP85-181P and EPP85-181RDB)		Antiproliferative and proapoptotic effect	Affecting ERK signal transduction pathway	[119]
	In vitro (A Paca-2, BxPC-3, AsPC-, HPAC and PANC-1)	6.25, 12.5, 25, and 50 μM	Cancer cell death	Restricted RAGE in pancreatic cancer cell	[120]
Apigenin	In vitro (BxPC-3)	100, 200, and 400 μmol/L	Death of cancer cells	The G2/M cell cycle was halted and Bcl-2 expression was lowered, while Bax gene expression was increased.	[121]
	In vitro (AsPC-1, CD18, MIA PaCa2, and S2-013)	6.25–100 μM	Cancer cell death	DNA synthesis washindered, the G2/M cell cycle was arrested, and levels of cyclin A, cyclin B, cdc2, and cdc25 were all reduced.	[132]
Sulforaphane	In vitro (AsPC-1, BxPc-3, Capan-1 and MIA-PaCa2)	10 μM	Cell proliferation inhibited	NF-kB binding was prevented, downregulating apoptosis inhibitors and inducing apoptosis	[123]
	In vitro and in vivo (Human PC stem cells and mice)	10 μM	Cell proliferation inhibited	Blockade of hedghehog pathway, smo, Gli1, Gli2, Nanong, Oct-4, VEGF and PDGFRα	[124]
Alantolactone	In vitro and in vivo (BxPC-3, AsPC1, PANC-1 and BALB/c mice)	1.98 and 2.15 μM	Cancer cell death	Downregulation of STAT3 signaling pathway	[125]
	In vitro and in vivo (MIA PaCa-2, PANC-1 and BALB/c mice)	10 μM	Apoptosis	CTSB/CTSD protein activity and expression were inhibited and TFEB was reduced.	[126]
Thymoquinone	In vivo (Nude mice)	5 and 20 mg/kg	Apoptosis	Reduced XIAP and MMP-9 expression	[127]
Lycopene	In vitro (PANC-1)	0.25 and 0.5 μM	Reduced cancer cell growth	Decreased ROS level and NF-kB expression and increased caspase-3 and Bax to Bcl-2 ratio	[128]
Luteolin	In vitro (PANC-1, CoLo-357 and BxPC-3)	40 μmol/L	Induces programmed cell death	Increased Bax while reducing Bcl-2 protein and increasing caspase-3	[129]
Caffeic acid	In vitro (BxPC-3 and PANC-1)	10 µg/mL	Induces apoptosis	Mitochondrial dysfunction and activation of caspase-3/caspase-7	[130]
	In vitro and in vivo (PANC-1 and BALB/c mice)	5 μg/mL	Reduces cancer cell progression	Inhibited the expression of Twist 2 and vimentin	[131]

## Data Availability

Not applicable.
